# Illnesses Encountered during Medical Volunteering in Takeo Province, Cambodia

**DOI:** 10.3390/medicina56010030

**Published:** 2020-01-10

**Authors:** Hye-Yoon Lee, Sung Hun Choi, Jae Suk Rim, Ho-Kyung Lim, Young Soo Heo, Jeonghee Shin, Rinet Aieng, Chai Hong Rim

**Affiliations:** 1 Division of Breast and Endocrine Surgery, Department of Surgery, Korea University Ansan Hospital, Gyeonggido 15355, Korea; heygemma@korea.ac.kr; 2 Department of Internal Medicine, Barun Mind Hospital, Daejeon 35220, Korea; sernote@gmail.com; 3 Department of Oral and Maxillofacial Surgery, Korea University Guro Hospital, Seoul 08308, Korea; jaesrim@korea.ac.kr (J.S.R.); ungassi@naver.com (H.-K.L.); 4 Department of Dermatology, Guro Hospital, Korea University College of Medicine, Seoul 08308, Korea; ys644@hanmail.net; 5 Department of Pediatrics, Guro Hospital, Korea University College of Medicine, Seoul 08308, Korea; sourerkr@korea.ac.kr; 6 Tokyo Beauty Clinic, Chomkamon, Phnom Penh 12300, Cambodia; aiengrinet@gmail.com; 7 Department of Radiation Oncology, Korea University Ansan Hospital, Gyeonggido 15355, Korea

**Keywords:** medical volunteering, Cambodia, hypertension

## Abstract

*Background and Objectives*: Medical volunteering seeks to meet the clinical needs of underserved areas, but has been criticized for difficulties in addressing local health issues and resultant lack of sustainability. Our team has visited rural Cambodia annually since 2012. This study reports the illnesses encountered during the recent mission and share our experiences to improve the efficiency of medical volunteering. *Materials and Methods*: Infrastructure, such as public electricity or water, was unavailable, hence most medical care and records were hand-performed. We categorized (1) primary diagnoses (chief complaints) by duration of symptoms, and (2) primary and secondary diagnoses (illnesses that were not related to the chief complaint) by severity of illness since patients commonly reported multiple symptoms. Blood pressure and anthropometric values were also checked and analyzed. *Results*: We encountered 317 adult and 141 pediatric patients. Among adults, 61.3% had persistent chronic (>6 month) symptoms of their chief complaints. The commonest diagnoses of chronic symptoms were musculoarthritis (31.5%) and gastroesophageal reflux disease and/or gastritis (21.7%). Hypertension and/or cardiac problems were relatively common among males (13.6%). The most common diagnosis among the severest cases (specialized or intensive care recommended) was cardiac problems (14.8%), often with abnormalities in sonography or electrocardiogram. For children, the overwhelming majority of diagnoses were related to acute symptoms and low severity, and approximately half were cases of the common cold. Commonly prescribed drugs were antacids or mucosal protectors (31.3%), Non-steroidal anti-inflammatory drugs (NSAIDs) or other painkillers (27.6%), and antiparasites (17.7%) in adults, and NSAIDs (44.7%) and antiparasites (23.2%) in children. Among adults, 32.7% were diagnosed with hypertension, and body mass index (*p* = 0.003) and age (*p* < 0.001) were both correlated with hypertension and its grade. *Conclusions*: Our study offers practical help to volunteer health workers planning to visit Southeast Asia.

## 1. Introduction

Medical volunteering is an active means of aiding local communities in developing countries to fulfill unmet medical needs. This practice has been increasing, and over 6000 medical mission trips are conducted by US medical practitioners annually [1]. These missions provide people in the developing world with a sense of solidarity and self-confidence. Moreover, for the participating medical professionals, who mostly engage in highly specialized daily practices, these missions are a reminder of their humane role as physicians [2,3].

There has also been considerable criticism of these missions despite their fundamental merits, primarily because volunteers are not full time but part time and because more than 70% of medical volunteering is short-term (less than four weeks) [4]. Short medical volunteering is mostly criticized for being too self-serving, meaning that the values and interests of the participating medical practitioners are put before the local community’s medical needs. With a lack of sustainability, only temporary treatments can be performed and the root cause cannot be resolved. Furthermore, non-medical expenses for medical trips (e.g., travel expenses, insurance, and acceptable levels of food and accommodation) may make it difficult to maintain cost-effectiveness [1,5].

A small medical volunteering team in Korea, “Near to Sky”, was organized by the authors of this study. Our team has provided short-term medical services to Cambodia’s rural areas once a year from 2012 to 2019 and treated 300 to 600 patients each year [6]. We suffered from a lack of practical literature for reference during the early years of our service. Many existing studies had a semantic perspective. Most of these studies were based on experiences in Africa or Latin America, and the literature on tropical Asia was even scarcer [4,6].

Rovers et al. [7] suggested that volunteering can also have a research component that provides locally relevant results and, based on this idea, our team published a previous report of our experiences [6]. In February 2019, we also went on a mission to a rural area in Cambodia, which was a new destination. The mission in 2019 was attended by five specialists, the most so far, and added medical equipment, such as portable ultrasound, electrocardiogram, and blood pressure monitor machines.

We believe that our experiences can be helpful in identifying the trends of illness in rural Cambodia. In addition, by combining the knowledge gained from our experiences with those of other medical volunteers, we might more easily determine the unmet medical needs of rural Cambodians and promote the sustainability of the measures taken to fulfill those needs. While the level of treatment and diagnoses did not reach modern medical standards, we report our experiences below.

## 2. Materials and Methods

### 2.1. Overview of Cambodia and the Studied Region in Takeo Province

The Kingdom of Cambodia is located in the southern part of the Indochina Peninsula and it has an area of 180,000 square kilometers and a population of about 16 million. The country became independent from France in 1953. Between 1975 and 1979, approximately two-million Cambodians were killed during the Khmer Rouge regime, and a significant number of health care providers also died [8,9]. The physician density is 0.17 per 100,000 people, which is in the lowest quartile among the world’s countries [6]. According to a World Health Organization report, the median age of the entire population is 24 years, and the population aged over 60 years is only 8%. The majority (80%) of the population lives in non-urban areas. The maternal mortality ratio is 1200 per 100,000 live births, and the leading causes of death are heart disease (10.1%), tuberculosis (9.6%), stroke (8.7%), and lower respiratory infection (7.8%) [8]. In terms of economics, gross national income per capita has been steadily increasing; it was about 2500 United States dollars in 2010, but it reached 3870 United States dollars in 2018, with an annual growth rate of 7.5% [10].

Takeo Province, which is one of the 24 provinces of Cambodia, is located in the southernmost part of the country. The province is bordered on the south by Vietnam. There are approximately 1100 villages in Takeo Province and the population is estimated to be 900,000, of whom 90% work in or live by farming [8]. This study was set in the Boeng Tranch Khang Tbong commune in the province and our mission clinic was located in the teachers’ office of a primary school.

### 2.2. Volunteer Activities

Our team has been conducting medical mission trips annually since 2012, and each mission has served approximately 300 to 600 patients for about a week. Based on our past experiences, we attempted to bring together various specialists and local medical providers to conduct efficient missions and cope with every possible situation. Korean volunteers included an internal medicine specialist, a general surgeon, two dentists specializing in oromaxillary surgery, a nurse, a photographer, and a radiation oncologist who has directed the last seven missions. Among Cambodians, a local doctor also participated as a volunteer, and three medical college students participated as translators and medical advisors.

Public electricity was provided at the school, but it was unstable and would often be interrupted with the use of high-powered electrical devices, such as a dental sterilizer. Tap water was not provided and drinking water was purchased in advance from a nearby town (Supplement Figure S1). Observations and data were recorded by hand in medical charts that had been developed based on our previous experiences (Supplement Figure S2). The medicine list used as a reference is provided in a supplemental table in our previous article [6]. We prepared a portable blood pressure monitor, echocardiogram, and ultrasound because cardiovascular disease is the leading cause of death in Cambodia.

### 2.3. Data Collection and Assessment

Adult patients were routinely assessed for age, height, body temperature, and blood pressure. In pediatric patients, age, weight, and body temperature were assessed in all possible cases, and blood pressure was not measured. Primary diagnosis was based on chief complaints, and secondary diagnosis was determined when secondary complaints were not associated with chief complaints because a large number of patients complained of multiple symptoms. The primary diagnoses, based on chief complaints, were classified as acute (lasting one month), subchronic (lasting 1–6 months), and chronic (lasting six months or more). In order to evaluate the severity of each case, three specialists discussed and categorized the severity of each case (while considering both primary and secondary diagnoses, and other symptoms whenever possible) into three stages by majority rule: (1) short-term care needed; (2) long-term care needed; and, (3) specialized or intensive care recommended. The above two assessments, related with symptom duration and severity, were used to determine which illnesses were most seriously harmful to local health. Blood pressure was assessed and categorized according to the Seventh Report of the Joint National Committee on Prevention, Detection, Evaluation, and Treatment of High Blood Pressure (Stage 1: systolic blood pressure 140–159 and/or diastolic blood pressure 90–99; stage 2: systolic blood pressure ≥160 and/or diastolic blood pressure ≥100) [11]. With regard to dental assessment, detailed history was not taken, owing to a lack of sufficient manpower, and relevant illnesses and actions being performed were briefly checked.

Most data are reported while using descriptive statistics. However, the Jonckheere–Terpstra test was used to analyze the relationship between hypertension and body mass index (BMI) or age, and a linear-by-linear association method was used to determine the relationship between hypertension and gender. All of the statistical analyses were performed while using SPSS version 23.0 (IBM Corp., Armonk, NY, USA).

The Institutional Review Board of Ansan Hospital, Korea University Medical Center, Gyeonggi-Do, Korea approved this study (IRB No: K20191796001, approved at 5 November 2019).

## 3. Results

During the mission, we encountered a total of 317 adult patients (216 females and 101 males) and 141 pediatric patients (age under 18). Table 1 presents the age and anthropometric values, including height and weight, and the BMI of all the patients was calculated.

Among all of the adults who were available for blood pressure measurement, 100 of 310 (32.3%) were categorized as having hypertension; 71 (22.9%) had grade 1 hypertension, and 29 (9.4%) had grade 2 hypertension. BMI and age were both significantly correlated with hypertension and its grades (*p* = 0.003 and <0.001, respectively). Gender showed a non-significant trend (*p* = 0.069), with fewer patients without hypertension and more patients with grade 2 hypertension among males. Table 2 summarizes the above results.

Among the patients who indicated the duration of the chief complaint, 61.3% had chronic (>6 month) symptoms, and acute and subchronic symptoms each accounted for approximately half of the remainder. Regardless of duration, musculoarthritis and gastroesophageal reflux disease (GERD) and/or gastritis comprised approximately two-thirds of all primary diagnoses. The most common primary diagnoses for acute (≤1 month) symptoms were common cold (17.4%) and musculoarthritis (17.4%), followed by GERD and/or gastritis (13.0%). The most common diagnoses for subchronic (one to six months) symptoms were musculoarthritis (31.8%) and GERD and/or gastritis (25.0%). Cystitis and/or vaginitis was a relatively common diagnosis among females (15.3%). The most common diagnoses for chronic (>6 month) symptoms were musculoarthritis (31.5%) and GERD and/or gastritis (21.7%). Among chronic illness, hypertension and/or cardiac problems were relatively common among males (13.6%). Other less common diagnoses included dermatitis, headache, pneumonitis, and/or chronic obstructive pulmonary disease, minor traumas, and mild psychiatric disorders, including insomnia and depression. We divided the primary diagnoses into two categories (acute with ≤1 month and chronic with >1 month) for children, because the majority (80.7%) of these diagnoses were related to acute chief complaints. Approximately half of the primary diagnoses related to acute chief complaints were of the common cold (54.3%), whereas GERD and/or gastritis (8.3%), rhinitis (7.1%), and pneumonitis (6.0%) were less frequently diagnosed. Common diagnoses of chronic chief complaints were sustained upper respiratory infection (20.0%) and GERD and/or gastritis (15.0%). Table 3 summarizes the above results.

Regarding to the severity of the cases, 68.3% of all diagnoses were “short-term care needed” cases, and the other two categories each made up half of the remainder. GERD and/or gastritis (26.9%) and musculoarthritis (25.9%) were the most common diagnoses for “short-term care needed” cases. Musculoarthritis (37.1%) was the most common diagnosis for the “long-term care needed” cases, but hypertension (14.3%) and pneumonitis and/or chronic obstructive pulmonary disease (11.4%) were also common. Diagnoses among “specialized or intensive care recommended” cases varied somewhat, and cardiac problems (14.8%, mostly diagnosed with sonography or electrocardiography), thyroid nodules (8.2%, diagnosed with sonography), possible Meniere’s disease or benign paroxysmal positional vertigo (8.2%), and psychologic disorder (6.6%, mostly insomnia or depression) were relatively common, apart from musculoarthritis (13.1%) and GERD and/or gastritis (11.5%). Regarding children, almost 90% of diagnoses were of “short-term care needed” cases and 45.1% of these cases were the common cold. Three out of four diagnoses of “long-term care needed” cases were malnutrition. Among nine diagnoses of “specialized or intensive care recommended” cases, five (55.6%) were otitis and/or tonsillitis and two (22.9%) were pneumonitis. Table 4 summarizes these data.

The most commonly prescribed medication was antacid or gastrointestinal protectors (31.3%), being commonly prescribed not only for gastritis but also to protect the stomach from drug irritation, followed by nonsteroidal anti-inflammatory drugs or other painkillers (27.6%) and antiparasites (17.7%). Among children, the most common drug used was nonsteroidal anti-inflammatory drugs (44.7%), followed by antiparasites (23.2%). Table 5 shows the most commonly used drugs among the adults and children by category.

Among the adult patients, 43 of 317 (13.5%) had been treated by a dentist. Cleansing or scaling was performed in 12 of 43 patients (27.9%), and tooth extraction was performed in 10 of 43 patients (23.3%). The major causes of extraction were deep or multiple caries for patients younger than 30 years old, and periodontitis for those who were older than the former. Among the children, 13 of 141 patients (9.2%) were referred to a dentist, and six of them (46.1%) underwent tooth extraction. The most common cause of reference was multiple or deep caries.

## 4. Discussion

When we first began medical volunteering in 2012, reference studies with practical information that could aid in actual preparation were lacking. Most of the studies on medical volunteering have analyzed the significance of the activities or investigated the health care aspects of missions from a broad perspective. Our team has been undertaking medical mission trips to rural Cambodia annually for the past eight years, and the goal of this study was to share our experiences, so that other volunteers and health care providers can take more effective approaches to fulfilling unmet local needs. Our study revealed the distribution of illnesses in the setting of medical volunteering in rural Cambodia and comprehensively showed clinical needs by analyzing the illness distribution by the case severity and duration of symptoms. These methods can serve as a tool in future research on medical volunteering experiences. Our data also showed that nearly one-third of adult patients have hypertension, which suggests that the prevention of hypertension should be policy-driven and medical volunteers should also be actively involved in management.

The present study found that musculoarthritis and GERD and/or gastritis were the most common diagnoses, which corresponds with the findings of our previous report [6]. The findings of both studies are in line with those of a large interview-based study [12], which showed that physical injury and stomachache are among the top ten illnesses reported. Given that most cases of these two major illnesses had chronic symptoms in our study, it is thought that they are related to the lifestyle of tropical Asian rural areas, rather than to specific etiologies. Most patients with musculoarthritis complained of pain associated with farming. Some patients complained of sequelae caused by inadequate treatment of injuries, often from traffic accidents or accidents at work. Unfortunately, this illness cannot be resolved with short-term intervention; however, education on stretching, proper rest, or first aid in cases of injury can be helpful in the long term. Gastrointestinal illness might be reduced if enough clean water is provided to cleanse food and refrigerators are made available to keep the foods fresh [6]. Control of hypertension and screening for heart disease for high-risk populations is also important when considering the severity of the illnesses in our study.

The findings of the present study are similar to those of our previous report and the interview study [6,12] that common colds or upper respiratory infections make up the majority of childhood illnesses. Living in simple timber houses and staying outside during most of the day, as in rural areas of Cambodia, might be a risk factor for dengue fever transmission by mosquitoes [13]. A study in two rural hospitals in Cambodia reported that *Haemophilus influenzae* and *Streptococcus pneumonia* are the most common etiology of pneumonitis [14]. Immunization and protection against pests are necessary, given that acute respiratory infection is the leading cause of mortality in children [8]. It is advisable to prepare antiparasite drugs for medical volunteering since illnesses due to parasites are not uncommon in rural Cambodia [15,16]. Encouragingly, we have seen an increase in the number of children receiving antiparasite drugs at school throughout our annual missions.

Ischemic heart disease and stroke are the first and third leading causes of death, respectively, in Cambodia [8], and they are both closely related with chronic hypertension. In a large-sized interview-based study in rural Cambodia, the serious illness most commonly reported by locals was hypertension [12]. The prevalence of hypertension is more serious in poor and rural areas; in a previous study, the incidence was 12% in a semi-urban community near Siem Reap, but 25% in a rural community of Kampong Cham Province [17]. In our study, it was 32.3%, which suggests both its seriousness and possible diversity among regions. BMI and male gender were related with the incidence and severity of hypertension in our study, which corresponds with the findings of previous studies [18,19]. In local communities of Cambodia, unmanaged hypertension can cause serious complications, such as paralysis or sudden death, damage the workforce, and cause financial burden, hence distressing patients and their families [20].

Our experiences so far indicate that only a few patients are on medication, and the majority of these patients take drugs intermittently from the private drug stores nearby. We rarely encountered patients who received continuous checkups. Many medical volunteering teams were reluctant to carry medicine for chronic diseases, because continuous checkup and medication guidance seemed difficult. However, most of the rural people in Cambodia are aware of the seriousness and necessity of medication for hypertension (e.g., they commonly hear about sudden deaths or paralysis of acquaintances) and they usually do their best to continue medication, as by following a previous prescription from the public clinic (where the relatively poor rural dwellers can be treated without cost, but with long journeys and waits) in private drugstores [20]. Therefore, we recommend that medical volunteers prescribe hypertension medication if possible, while informing patients that continuous medication is necessary, rather than being reluctant to prescribe, owing to difficulties in following up. Oral clonidine and beta-blockers, for which possible rebound hypertension has been known, might be avoided [21,22], and prescriptions should be made simply so as to be understandable by locals with the help of local translators, so they can be continued with later visits to local drugstores or volunteering clinics.

In recent decades of Cambodia, major health indicators, such as life expectancy and maternal mortality, have significantly improved [9,23]. However, the common illnesses in the rural area have not changed much; as compared to the surveillance study performed in 2006–2007, the pattern and seriousness of illnesses are mostly similar to those in the present and our previous studies [6,12]. According to our experiences in three villages in different regions, a majority of illnesses seems to be affected by lifestyle. The most serious illness in the first village in Cham Lak Province was vaginitis and/or cystitis, being probably caused by contamination of the water source. The whole village used the same water source, a lake, for any use, including laundry, bathing, and even cooking. The most common illness was musculoarthritis, which was related to farming labor in the second village in Khsoem Province, and the third village, in Takeo Province, where the main water sources were town wells [6].

Therefore, we suggest that education and improvement of infrastructure, as well as medical assistance, can indeed help the local people. By assisting in electric installations, such as refrigerators, gastrointestinal illnesses will be much reduced. Domestic health care workers specializing in physical therapy should be trained to help people suffering musculoarthritis from the hard farming labor. Building wells and supplying basic hygiene tools, such as soaps, toothbrushes, and toothpastes, can be highly beneficial. In our experience, most local coordinators (usually nurses or medical students) have considerable experience in providing health education for local residents. Volunteers from developed countries can help to sharpen their medical expertise or support resources in need. Additionally, we believe that significant numbers of people need help from mental health care providers. Many patients suffered from serious insomnia or depression, probably owing to their chronic illnesses and poverty. However, there is no system or presence of specialists to provide appropriate care. Mental illnesses are regarded as symptoms of the weak and, sometimes, the patients are discriminated against in their society [24]. Literally, the rural area of Cambodia is no man’s land from the perspective of psychology. Hence, the participation of mental health specialists in such missions will be of great help for the assessment and care of problems and educating local health care providers.

Ultimately, professionals participating in volunteering services should support the locals’ own and sustainable care as well as provide medical help by collaborating with local institutions and providing education to local health providers. However, those goals are difficult to be achieved only with several short-term missions. The “triple win” ideology espoused by Logan et al. [25] is a good model for realizing this: Practitioners can achieve self-actualization and avoid career burnout by providing healthcare professionals with extended leave for humanitarian work. The local communities can benefit with timely expert care and professional development opportunities. Employers themselves will have the advantage of improving employee performance and reducing monetary losses to compensate for the burnout of employees. With these in mind, medical volunteers should strive to optimize the provision of services in a long-term perspective.

The main limitation of this study is that the diagnoses are mostly symptomatic and lack the ability to identify detailed etiologies. In the setting of short-term volunteering, mostly heading to rural areas of low-income countries, the levels of diagnosis and treatment are far below modern standards. Medical records are usually handwritten in various formats, and considerable effort is required to collect them. For those reasons, medical volunteers are reluctant to conduct research that is based on their experiences and grow tired of providing superficial services that do not really meet local needs. However, the results of our study suggest that the majority of clinical needs can be met with primary care, not with high-end surgical techniques or up-to-date medicines. For residents of rural areas in Cambodia, the provision of sustainable care for common illnesses is necessary, rather than having someone arrive carrying large bags and offering difficult surgery. In our view, this is the role that hospitals in local-based cities should play. During our missions, we often heard from locals that other service teams had been in nearby villages just a few days earlier or even that other teams were volunteering in neighboring villages at the same time we were active. Sharing data on medical volunteering experiences will bring us a step closer to addressing the unmet needs of locals. Furthermore, this sharing of data might overcome the issue of sustainability in a broad sense if many teams can provide effective and tailored services and, hence, continue their activities.

## 5. Conclusions

Our study, which was based on a recent medical mission trip to Takeo Province, showed that GERD and/or gastritis, and musculoarthritis often related to farming, were the most common illnesses among locals. The majority of illnesses were related to long-term symptoms. Nearly one-third of adult patients were diagnosed with hypertension, and cardiac problems were the most common among the severe illnesses. The prevalence of hypertension in rural areas should be more actively controlled, and medical volunteers are recommended to take part in treatment. Researching and sharing the experience of volunteering is expected to aid services in meeting local clinical needs, and locals in receiving better and sustained health care.

## Figures and Tables

**Table 1:** Anthropometric characteristics of patients.

Patient Characteristics	Females (*n* = 216)	Males (*n* = 101)	Children (*n* = 141)
Age	53.5 (19–87)	53.5 (19–87)	10 (0.5–17)
Weight (kg)	52.1 (30.9–77.9)	57.4 (37.6–89.0)	23.6 (3.4–72.5)
Height (m)	1.54 (1.35–1.74)	1.65 (1.47–1.80)	
BMI	22.0 (14.4–33.5)	21.5 (14.9–29.5)	

BMI—body mass index; Data are presented as median (range).

**Table 2:** Hypertension according to BMI, age, and gender.

Patient Characteristics	No HTN (*n* = 210)	Grade 1 (*n* = 71)	Grade 2 (*n* = 29)	*p*-Value
	mean (95% confidence interval)			
BMI	21.7 (21.2–22.2)	22.8 (21.9–23.7)	22.9 (21.5–24.3)	0.003 ^†^
Age	49.7 (47.6–51.7)	55.5 (52.0–59.1)	58.7 (52.9–64.4)	<0.001 ^†^
	percentile among patients			
Females (*n* = 211)	70.6%	21.8%	7.6%	
Males (*n* = 99)	61.6%	22.7%	13.1%	0.069 ^‡^

BMI—body mass index; HTN—hypertension. ^†^ Jonckheere-Terpstra test. ^‡^ Linear-by-linear association.

**Table 3:** Primary diagnosis categorized by chronicity of chief complaints.

Duration of Chief Complaints			
Acute (≤1 month, 46 Cases)	Subchronic (1–6 months, 44 Cases)	Chronic (>6 months, 143 Cases)	(N/A 84 Cases)
*Adults*			
common cold (17.4%)	musculoarthritis (31.8%)	musculoarthritis (31.5%)	
musculoarthritis (17.4%)	GERD and/or gastritis (25.0%)	GERD and/or gastritis (21.7%)	
GERD and/or gastritis (13.0%)	cystitis and/or vaginitis (15.3%, female)	HTN and/or cardiac problem (13.6%, male)	
*Children*			
(≤1 month, 84 cases)	(longer than 1 month, 20 cases)		
common cold (54.8%)	sustained URI (20.0%)		
GERD and/or gastritis (8.3%)	GERD and/or gastritis (15.0%)		
rhinitis (7.1%)			
pneumonitis (6.0%)			

GERD—gastroesophageal reflux disease; HTN—hypertension; N/A—not applicable; URI—upper respiratory infection.

**Table 4:** Common illnesses categorized by severities (including primary and secondary diagnoses).

Severity of Illnesses			
Short-Term Care Needed	Long-Term Care Needed	Specialized or Intensive Care Recommended	
*Adults*			
(200 cases, 282 diagnoses)	(52 cases, 70 diagnoses)	(45 cases, 61 diagnoses)	(N/A 20 cases)
GERD and/or gastritis (26.9%)	musculoarthritis (37.1%)	cardiac illness (14.8%)	
musculoarthritis (25.9%)	hypertension (14.3%)	musculoarthritis (13.1%)	
headache (9.2%)	GERD and/or gastritis (12.9%)	GERD and/or gastritis (11.5%)	
common cold/URI (5.3%)	pneumonitis and/or COPD (11.4%)	thyroid nodule (8.2%)	
cystitis (4.6%)		rule out Meniere’s or BPPV (8.2%)	
dermatitis (3.2%)		psychologic disorder (6.6%)	
*Children*			
(115 cases, 122 diagnoses)	(4 cases, 4 diagnoses)	(8 cases, 9 diagnoses)	(N/A 14 cases)
common cold (45.1%)	malnutrition (75%)	otitis and/or tonsillitis (55.6%)	
GERD and/or gastritis (9.0%)		pneumonitis (22.9%)	
headache (6.6%)			
dermatitis (5.7%)			
rhinitis (5.7%)			

BPPV—benign paroxysmal postural vertigo; COPD—chronic obstructive pulmonary disease; GERD—gastroesophageal reflux disease; N/A—not applicable; URI—upper respiratory infection.

**Table 5:** Commonly prescribed medications.

Type of Medications	Prescription Cases	(%)
*Adults*		
Antacids or GI mucosal protectors	232	31.3%
NSAIDs/painkiller	205	27.6%
Antiparasites	131	17.7%
Anticough or mucolytics	53	7.1%
Antibiotics	36	4.9%
Others	353	11.5%
	742	
*Children*		
NSAIDs	85	44.7%
Antiparasites	44	23.2%
Electrolyte supplements	13	6.8%
Antibiotics	11	5.8%
Antacids or GI protectors	10	5.3%
Others	27	14.2%
All prescription cases	190	

GI—gastrointestinal; NSAID—nonsteroidal anti-inflammatory drugs.

## Supplementary Materials

The following are available online at https://www.mdpi.com/1010-660X/56/1/30/s1, Figure S1: Overview of during the mission. We used teacher’s tables to meet the patients in the teacher’s lounge of the school. On the left, a large tea-table covered with sheets was served as a bed for portable sonographic exam. Figure S2: Patient chart used in ‘Near to sky’ missions. It is designed to quickly get information from the patients commonly encountered in medical volunteering, and written in easy English for use of international doctors and local interpreters. Chai Hong Rim, who is an author of the present study and designed the chart, happily agrees use of the chart freely for medical volunteering.
