# Supplementary material for: Illnesses Encountered during Medical Volunteering in Takeo Province, Cambodia
Source: Medicina (Kaunas). 2020 Jan 10;56(1):30. doi: 10.3390/medicina56010030 (PMC7023312; doi:10.3390/medicina56010030)
Supplement: Supplementary file 1 [file medicina-56-00030-s001.pdf]

Supplement materials

The possibly identifiable persons in S1 figures are Dr. SH Choi AND HY Lee, the authors of the present study, who declared full agreement of publication of images.

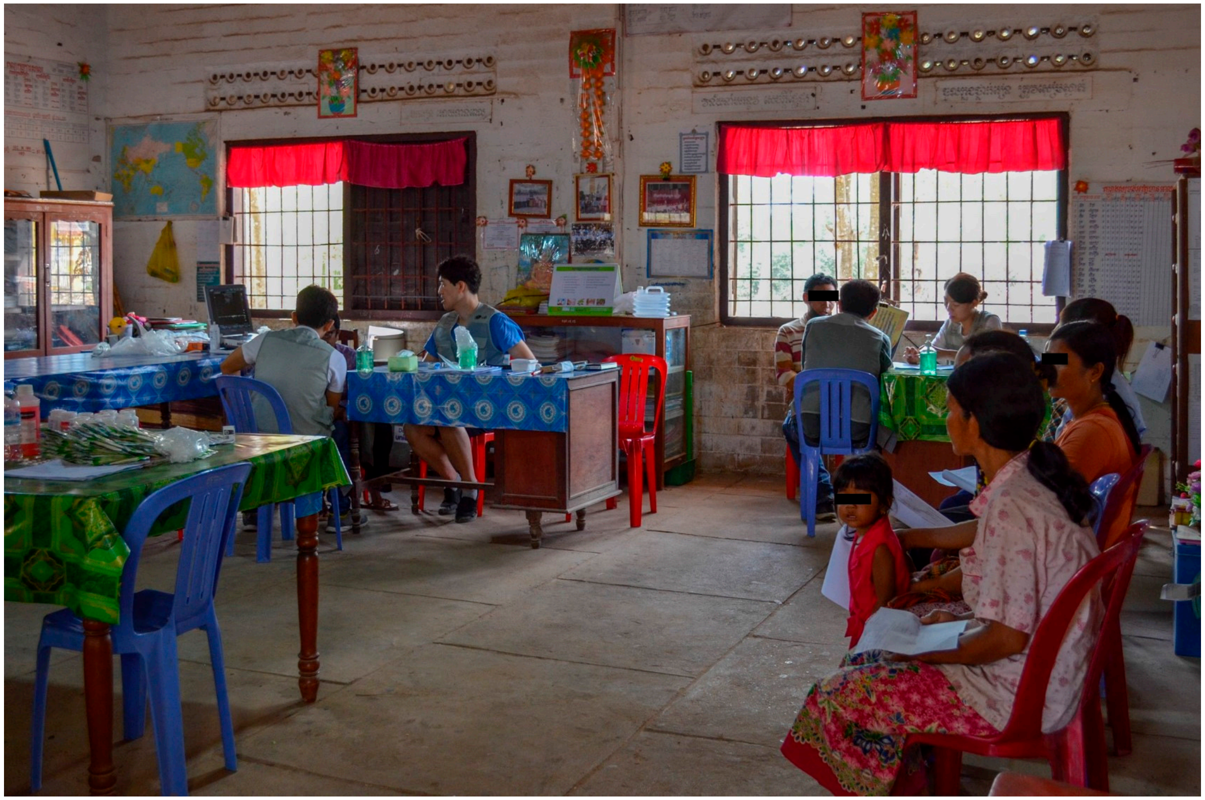

Supplement Fig 1. Overview of during the mission. We used teacher's tables to meet the patients in the teacher's lounge of the school. On the left, a large tea-table covered with sheets was served as a bed for portable sonographic exam.

Patient Chart

Name : **ID :**

Age/Sex :

Weight: Blood Pressure: /
Height:

Chief Complaints (Onset);
Detailed Descriptions;

Anal itching or did parents see worms or eggs in children's stool? (Y / N)
Did the child take anthelmintic drug in recent 6 months ?
Does the patient have any possibility of pregnancy? (Y / N)

Common cold & Respiratory Sx.
Body temperature : `C
Cough/Sputum/Rhinorrhea (/ /)
Sputum color (Yellow / White / Blood-tinged/ Green)
Headache/Chilling/Myalgia (/ /)

GI Sx.
Nausea/Vomiting/Diarrhea/Constipation (/ / /)
Pain at where? (RUQ/ LUQ/ RLQ/ LLQ/ central/ lower abdomen/ whole abdomen)
Blood in stool?

Genitourinary Sx.
Frequent urine/ Urinal pain/ Bloody urine (/ / /)
Vaginal discharge / color (/) Pruritis ( )
Bad smell? (Smells like what?)

Dermatologic Sx. & Arthralgia
Hx. of insect bite?
Hx. of working in dirty water?

Pain at where?
What kind of work the patient do?

Other symptoms.

Doctor's note

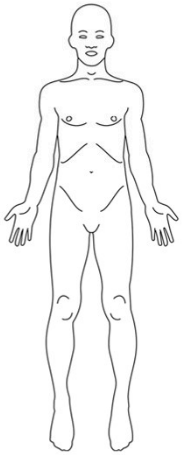

Dx.

Prescription :

Supplement Fig 2. Patient chart used in 'Near to sky' missions. It is designed to quickly get information from the patients commonly encountered in medical volunteering, and written in easy English for use of international doctors and local interpreters. Chai Hong Rim, who is an author of the present study and designed the chart, happily agrees use of the chart freely for medical volunteering.
